# Switched alternative splicing events as attractive features in lung squamous cell carcinoma

**DOI:** 10.1186/s12935-021-02429-2

**Published:** 2022-01-05

**Authors:** Boxue He, Cong Wei, Qidong Cai, Pengfei Zhang, Shuai Shi, Xiong Peng, Zhenyu Zhao, Wei Yin, Guangxu Tu, Weilin Peng, Yongguang Tao, Xiang Wang

**Affiliations:** 1 Department of Thoracic Surgery, Second Xiangya Hospital, Central South University, Changsha, 410011 China; 2 Hunan Key Laboratory of Early Diagnosis and Precise Treatment of Lung Cancer, Second Xiangya Hospital, Central South University, Changsha, 410011 China; 3 Xiangya School of Medicine, Central South University, Changsha, 410008 China; 4 Key Laboratory of Carcinogenesis and Cancer Invasion, Ministry of Education, Department of Pathology, Xiangya Hospital, Central South University, Hunan, 410078 China; 5 NHC Key Laboratory of Carcinogenesis (Central South University), Cancer Research Institute and School of Basic Medicine, Central South University, Changsha, 410078 Hunan China

**Keywords:** Alternative splicing, Lung squamous cell carcinoma, Machine learning algorithms, Splicing switch, Biomarkers

## Abstract

**Background:**

Alternative splicing (AS) plays important roles in transcriptome and proteome diversity. Its dysregulation has a close affiliation with oncogenic processes. This study aimed to evaluate AS-based biomarkers by machine learning algorithms for lung squamous cell carcinoma (LUSC) patients.

**Method:**

The Cancer Genome Atlas (TCGA) database and TCGA SpliceSeq database were utilized. After data composition balancing, Boruta feature selection and Spearman correlation analysis were used for differentially expressed AS events. Random forests and a nested fivefold cross-validation were applied for lymph node metastasis (LNM) classifier building. Random survival forest combined with Cox regression model was performed for a prognostic model, based on which a nomogram was developed. Functional enrichment analysis and Spearman correlation analysis were also conducted to explore underlying mechanisms. The expression of some switch-involved AS events along with parent genes was verified by qRT-PCR with 20 pairs of normal and LUSC tissues.

**Results:**

We found 16 pairs of splicing events from same parent genes which were strongly related to the splicing switch (intrapair correlation coefficient = − 1). Next, we built a reliable LNM classifier based on 13 AS events as well as a nice prognostic model, in which switched AS events behaved prominently. The qRT-PCR presented consistent results with previous bioinformatics analysis, and some AS events like ITIH5-10715-AT and QKI-78404-AT showed remarkable detection efficiency for LUSC.

**Conclusion:**

AS events, especially switched ones from the same parent genes, could provide new insights into the molecular diagnosis and therapeutic drug design of LUSC.

**Supplementary Information:**

The online version contains supplementary material available at 10.1186/s12935-021-02429-2.

## Introduction

Lung cancer is a worldwide medical problem and carries a heavy disease burden. At present, lung cancer is still a commonly diagnosed cancer in the world (11.4%) only second to breast cancer (11.7%), while its mortality rate ranks first among all malignant tumors (18.0%) due to its high aggressiveness and atypia [1, 2]. Primary lung cancer can be divided into small cell lung carcinoma (SCLC) and non-SCLC (NSCLC) according to the type of tumor cells [3]. NSCLC accounts for more than 80% of all lung cancer cases and has two predominant histological subtypes as lung adenocarcinoma (LUAD) and lung squamous cell carcinoma (LUSC) [4]. In the last decades, because of medical progress such as early screening, surgical techniques, and chemoradiation, the prognosis of lung cancer has been improved a lot [5]. However, the overall survival (OS) of LUSC is still poor and seems to be worse than that of non-squamous NSCLC [6]. So, it is of great significance to develop novel biomarkers and help the diagnosis and treatment for LUSC patients.

RNA alternative splicing (AS) is an essential process of post-transcriptional gene expression regulation, by which exons of pre-mRNAs could be retained or excluded in the mature messenger RNA (mRNA) isoforms [7]. Thanks to the advances in RNA-seq technologies, scientists discovered differential splicing of mRNAs [8]. In total, seven kinds of AS patterns—exon skip (ES), alternate donor site (AD), alternate acceptor site (AA), retained intron (RI), mutually exclusive exons (ME), alternate terminator (AT), and alternate promoter (AP)—happen in about 95% of human genes [9, 10]. In which, ES is the most common pattern in mammalian pre-mRNAs [11], and several kinds of drugs based on it have been approved [12]. AP and AT are a bit different from the other five generally-recognized basic modes, also making a great contribution to the mRNA and protein diversity [13]. Besides, sometimes AS also causes mRNA degradation by bringing in premature termination codons [14]. Because of the striking association with cancers, aberrant AS along with its regulation was even thought of as a novel cancer hallmark [15, 16]. The in-depth knowledge of “splicing code” would put us into a new era of disease diagnoses and treatments [17–19]. Meanwhile, the machine learning technology, which aims to construct predictive models from complex datasets based on underlying algorithms [20], offers a novel medium for the investigation of AS situations in LUSC. By monitoring the variations in splicing patterns, we have the potential to help making accurate diagnoses, determining treatment plans with the best response, and even intervening the regulation of splicing patterns.

In this article, we utilized several machine learning methods to identify differentially expressed AS events, explore a reliable classifier for the lymph node metastasis situation, and construct a prognostic model according to the TCGA-LUSC data. We emphasize the value of paired negatively-correlated AS isoforms derived from the same genes and verified some of their expression levels via qRT-PCR. We also performed functional enrichment analysis and investigated the correlations between the survival-related AS events and upstream splicing factors (SFs).

## Materials and methods

### Data collection and processing

The RNA transcriptome profiles with related clinical information of LUSC patients were obtained from the TCGA database (https://portal.gdc.cancer.gov/). AS events of LUSC were retrieved from TCGA SpliceSeq (https://bioinformatics.mdanderson.org/TCGASpliceSeq) [21]. Each AS event was named by combining its gene name, designated ID, and AS pattern (for example, UNG-24277-AP). The Percent Spliced In (PSI) analysis was performed for each exon. R package impute was utilized to impute those missing values.

To conduct a reliable analysis, AS events available in less than 30% of LUSC cases were excluded. Besides, AS events with an average PSI value ≤ 0.05 and standard deviation < 0.01 were excluded in this study. As for the survival analysis, we only included samples having at least 30 days of follow-up. Statistical analyses were performed with R software (version 3.6.2). Details regarding the machine learning algorithms, R packages, and codes involved in this study have been previously described [22].

### Identification of differentially expressed AS events in LUSC

As the normal samples only count about nine percent in the data set while the LUSC group makes up 91.1%, the *ovun.sample* function of R package ROSE was performed for normal samples to balance the data compositions. Then, Boruta feature selection was used to select AS events that could work in distinguishing LUSC samples from normal ones. Next, Spearman correlation analysis was applied to find AS events originated from the same gene, which reveals those AS events matter in the splicing switch of LUSC samples.

### Exploration of the LNM-related AS classifier

It is widely recognized that lymph node metastasis (LNM) is critical for determining the optimal treatment strategy and is an important prognostic factor for lung cancer patients. We utilized the Boruta algorithm to choose AS events linked with LNM. Following this, an ensemble learning technique—the random forest (RF)—was conducted to construct an AS event-based classifier with the smallest average error rate in a nested fivefold cross-validation. Besides, we checked the classification capacity of our selected classifier by calculating the area under the ROC curve (AUC) for the fivefold cross-validation.

### Construction of AS-based prognostic model

Random survival forest (RSF) is an adapted form of random forests, which mathematically builds binary recursive trees for all samples and aims to get the maximal survival difference across daughter nodes with the application of bootstrap methods and the log-rank splitting rule [23]. Whereas, Cox regression model, considering several involved variables and providing straightforward interpretations by hazard ratios, has been widely employed for survival analysis [24]. Here, we undertook the RSF model and Cox regression model independently, then selected survival-related AS events picked out by two models both. Next, the multivariate Cox regression was utilized to build an AS-based prognostic model. Furthermore, the Wilcoxon test was performed to explore the connection between such model and clinicopathologic factors, and a nomogram was developed based on its results. P < 0.05 was considered to be significant.

### Functional enrichment analysis

Kyoto Encyclopedia of Genes and Genomes (KEGG, https://www.genome.jp/kegg) is a knowledgebase of biomolecular pathways which have been automatically annotated, and Reactome (https://reactome.org) also offers a set of peer-reviewed reference pathways. For parent genes of survival-related AS events selected in the previous step, we analyzed potential functional pathways by KEGG and Reactome. ClueGO (version 2.5.5) of Cytoscape (version 3.7.2), a convenient plug-in designed for improved biological interpretation of several lists of genes [25], was utilized in such analysis.

### Construction of the splicing network

We downloaded 390 splicing factors (SFs) from SpliceAid 2 database (http://193.206.120.249/splicing_tissue.html) [26], then applied Univariate Cox analysis to choose prognosis-related SFs (P < 0.05). Spearman correlation analysis was performed to understand the relationship between these SFs and survival-related AS events with the inclusion criteria as P < 0.01 and |coefficient|> 0.2. Cytoscape (version 3.7.2) was used to present their correlations.

### Clinical tissue samples

With the approval of the ethics committee, primary lung cancer patients with LUSC who intended for surgical removal at the Thoracic Surgery Department of the Second Xiangya Hospital were involved in our study between February 2020 and August 2021. Fresh LUSC tissues and paired normal lung specimens were obtained and got preserved by liquid nitrogen with the informed consent of patients.

### RNA isolation and quantitative real‐time PCR (qRT-PCR)

Total RNA was extracted from ground tissues via Trizol reagent (Invitrogen) and reversely transcribed into cDNA via the SuperScript First Strand cDNA system (Invitrogen) according to the manufacturer’s protocols. The qPCR amplifications were performed in an Applied Biosystems Stepone Plus System (Applied Biosystems, Foster, USA) using an SYBR Green PCR Master Mix (HY-K0521, MCE, USA). The information of primers involved in qRT-PCR was listed in Additional file 1: Table S1. In order to verify the product, PCR products from randomly selected tissue samples were separated on 1.5% gels and then got observed on ChemiDoc XRS + imaging system (Bio-Rad, USA). Besides, the Sanger sequencing was performed (by Tsingke Biotechnology Co., Ltd.) and results were compared with the expected base sequence (through Chromas Version 2.6.6). For the qRT-PCR data, paired T-test was performed to obtain P values between the normal group and paired-LUSC group. The expression data of selected genes and PSI values of related AS events in TCGA were collected and analyzed by unpaired T-test to expound and prove the qRT-PCR results. GraphPad Prism 8.0 software (La Jolla, CA) was involved in plotting.

## Results

### Integrated AS event profiles in TCGA-LUSC cohorts

**Fig. 1:**
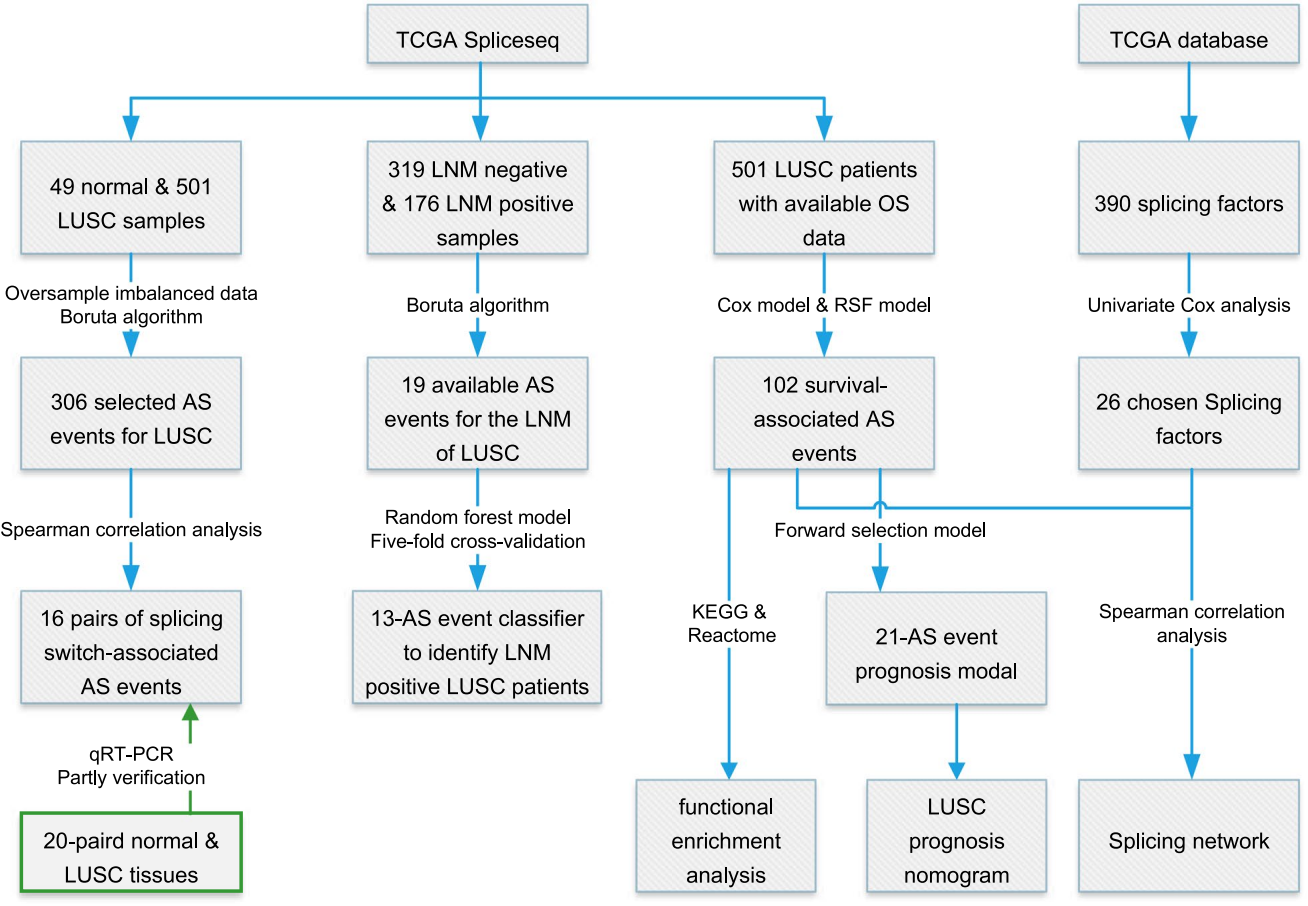
Schematic diagram of the overall study

**Fig. 2:**
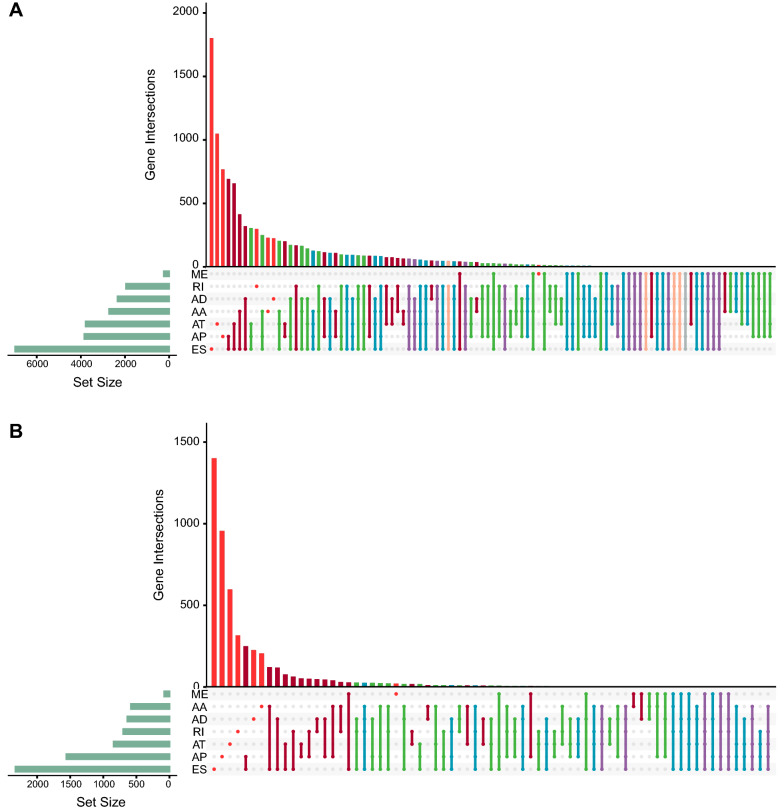
UpSet plots of AS events included in this study (**A**) and AS events after preliminary exclusion (**B**) presented in different splicing patterns. The exclusion criteria: AS events available in less than 30%, average PSI value ≤ 0.05, or standard deviation < 0.01). AS, alternative splicing

The schematic diagram of the overall study is presented in Fig. 1. Finally, AS data deriving from 49 normal samples and 501 LUSC samples, a total of 550, were left for the analysis of LUSC-specific AS events; 495 patients with available lymph node metastasis (LNM) data (319 negatives and 176 positives) were included for identification of LNM-related classifier; 501 patients with at least 30 days of follow-up were brought into the analysis of survival-related AS events (Additional file 2: Table S2). A total of 47,572 AS events coming from 10,727 genes were involved in this study, of which the top 3 splicing types make up about 78.1 percent. They are ES, AP, and AT with the proportion of 39.8%, 20.1%, and 18.2%, respectively. For the top 100 splicing patterns, 31 of them are combinations of three splicing types and a quarter are combinations of four splicing types (Fig. 2A). After filtering out AS events with less discrimination, 11,673 AS events spliced from 5,020 parent genes were included (Fig. 2B).

### Splicing switch-associated AS events

**Fig. 3:**
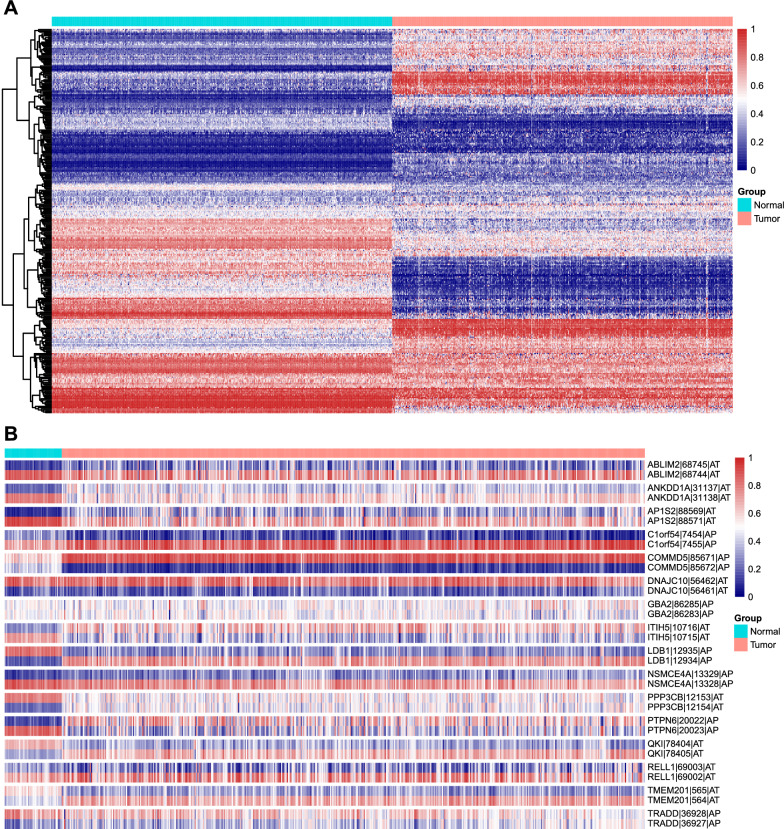
Heat maps in identifying AS events matter in the splicing switch of LUSC samples. **A** PSI levels of differentially expressed AS events between normal and LUSC samples after Boruta selection. **B** PSI levels of 16 pairs of completely negatively correlated AS events which expressed totally contrarily in normal and LUSC tissues

We oversampled normal cases to match with the amount of LUSC samples, then identified 306 AS events from 217 genes to distinguish these 2 tissue types via the Boruta algorithm (Fig. 3A, Additional file 3: Table S3). By analyzing AS events coming from the same parent genes, we found that 16 pairs of them have astonishing negative correlations as the intrapair correlation coefficient of every pair is − 1 (Fig. 3B): ABLIM2-68745-AT and ABLIM2-68744-AT, ANKDD1A-31137-AT and ANKDD1A-31138-AT, AP1S2-88569-AT and AP1S2-88571-AT, C1orf54-7454-AP and C1orf54-7455-AP, COMMD5-85671-AP and COMMD5-85672-AP, DNAJC10-56462-AT and DNAJC10-56461-AT, GBA2-86285-AP and GBA2-86283-AP, ITIH5-10716-AT and ITIH5-10715-AT, LDB1-12935-AP and LDB1-12934-AP, NSMCE4A-13329-AP and NSMCE4A-13328-AP, PPP3CB-12153-AT and PPP3CB-12154-AT, PTPN6-20022-AP and PTPN6-20023-AP, QKI-78404-AT and QKI-78405-AT, RELL1-69003-AT and RELL1-69002-AT, TMEM201-565-AT and TMEM201-564-AT, TRADD-36928-AP and TRADD-36927-AP. Our findings indicated the crucial role of some AS in the determination of cancer or normal tissues.

### Verification of important AS events

**Fig. 4:**
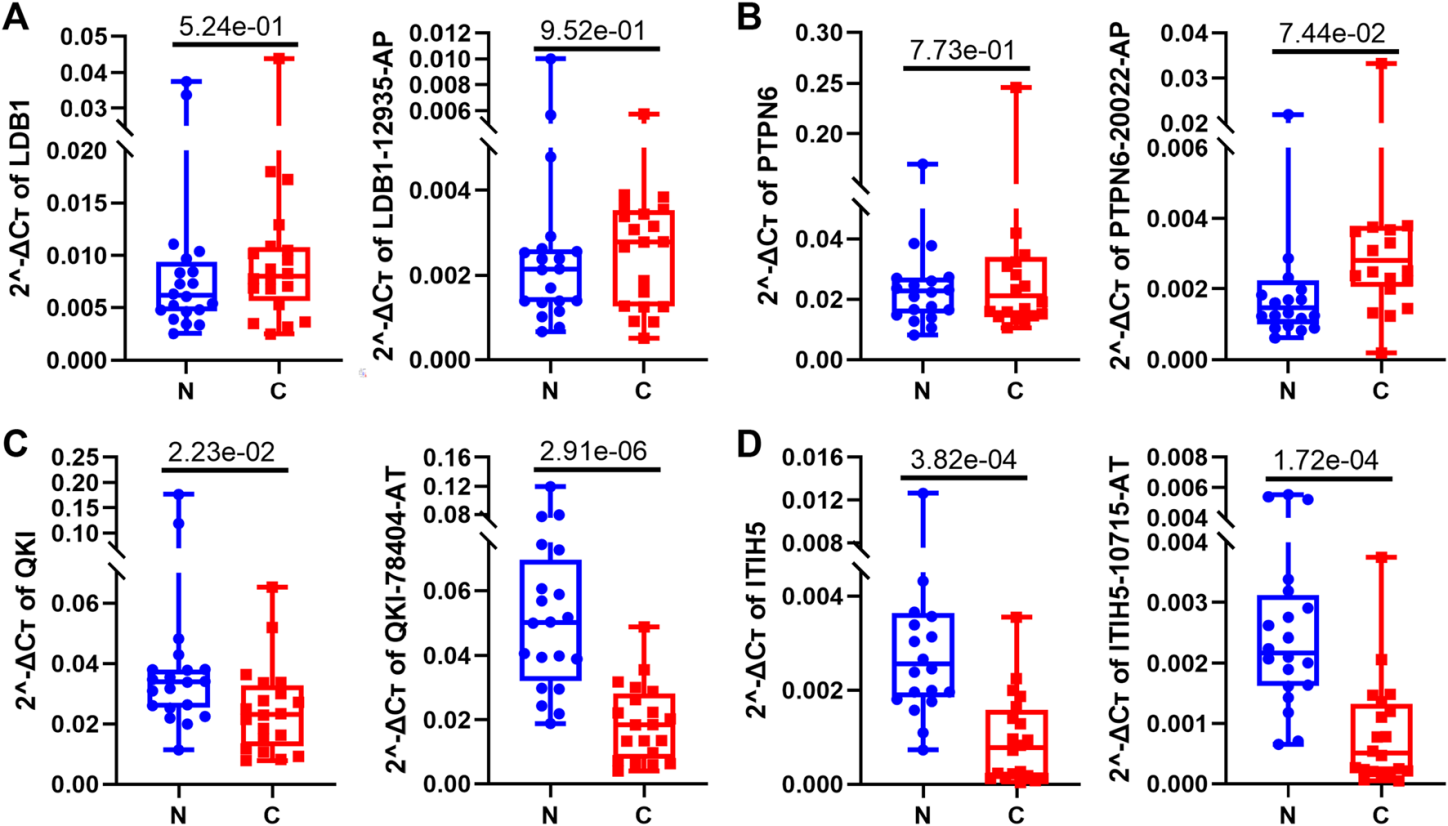
Verification of some genes or important AS events by qRT-PCR with 20 pairs of normal and LUSC tissues. Each dot represents an individual patient. Results are expressed as mean ± SEM. N, normal; C, cancer

In total, tissues from 20 LUSC patients were included in the cohorts (Additional file 4: Table S4), and baseline clinicopathological characteristics of LUSC data from TCGA and patient data utilized from our department were shown in Table 1. There is no statistically significant difference in gender, smoking history, or stages between the two groups, so we believe that our samples were accredited for validation. After analyzing the expression data of those splicing switch-associated AS events in LUSC and normal tissues by TCGA SpliceSeq (Fig. 3B, Additional file 5: Table S5), we found that alternate promoters (AP) of LDB1 and PTPN6, as well as alternate terminators (AT) of QKI and ITIH5, have the most obvious switch effects. We designed primers for some of their particular mRNAs and chose the primers which were verified by product sizes (Additional file 12: Figure S1A) and Sanger sequencing results. We noticed that the expression of ITIH5 in LUSC tissues is lower than that of normal lung tissues (p = 3.82e−04), whereas the level of ITIH5-10715-AT shows a better discrimination ability for them (p = 1.72 e−04). This phenomenon was also presented for QKI and QKI-78404-AT (p = 2.23e−02 and p = 2.91e−06, respectively). Besides, although there showed no statistical difference for the expression of LDB1, LDB1-12935-AP, PTPN6, or PTPN6-20022-AP between normal and LUSC tissues (Fig. 4, raw data in Additional file 6: Table S6). We also analyzed the expression of 4 genes in TCGA-LUSC data combined with related TCGA SpliceSeq data, which showed consistent change trends with our qRT-PCR results (Additional file 12: Figure S1B). The fold changes of LDB1 and PTPN6 are not so obvious compared with the other two, and the 20 pairs of tissues we tested may have some heterogeneity, which may explain such results. Furthermore, even though we only designed a primer for one kind of complementary AS events as well as the parent gene, we can speculate the expression of the other according to their “proportionally complementary expression relationship”. In a nutshell, we verified the expression of selected AS events successfully, and some of them showed better detection efficiency than their parent genes, supporting our bioinformatics analysis.

**Table 1:** The clinicopathological characteristics of LUSC data from TCGA and patient data utilized from our department

	TCGA data^a^	Test data	P value
n	501	20	
Gender (%)			0.795
Female	130 (25.9)	6 (30.0)	
Male	371 (74.1)	14 (70.0)	
Age (mean (SD))	67.20 (8.58)	53.90 (8.72)	< 0.001
Smoking history (%)			0.183
Nonsmoker	18 (3.7)	2 (10.0)	
Smoker	471 (96.3)	18 (90.0)	
Stage (%)			0.077
Stage I	244 (49.1)	16 (80.0)	
Stage II	162 (32.6)	3 (15.0)	
Stage III	84 (16.9)	1 (5.0)	
Stage IV	7 (1.4)	0 (0.0)	

^a^Some information of several patients was missed in TCGA

### AS-based LNM classifier

**Fig. 5:**
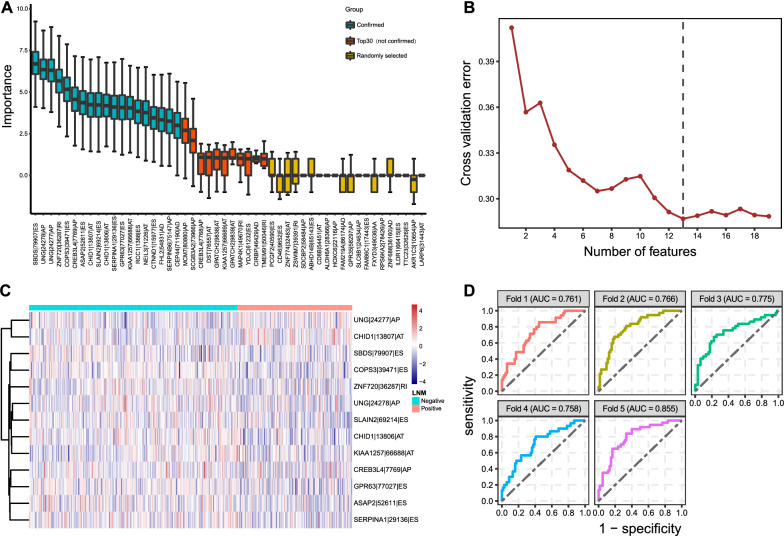
Exploration of the LNM-related AS classifier. **A** Z-score of the top 30 important AS events (top 19 of them were confirmed as important features, the other 11 were shown as red boxes) and other 20 randomly selected ones (in yellow) via the Boruta algorithm. **B** The mean cross-validation error regarding the number of AS events in the five-round five-fold cross-validation. **C** The heat map for PSI levels of AS events in the LNM classifier. The data were normalized via the R function *scale*. **D** ROC curves in identifying LNM status of LUSC patients with the classifier by the fivefold cross-validation. LNM, lymph node metastasis

In the Boruta algorithm, Z-scores were utilized to reflect the importance of features analyzed [22]. Here, we presented 30 AS events with the top Z-scores for LNM and the other 20 randomly chosen AS events in Fig. 5A, illustrating that the importance of the top 30 AS events is obviously higher than that of others. A dataset with 19 AS events spliced from 17 genes was selected for further exploration (Additional file 7: Table S7). The 495 patients with available LNM data were randomly assigned into the training set or the test set, and the average error in fivefold cross-validation was calculated. We found that a 13-AS event group could make the average rate to the minimum (Fig. 5B) so that we got an LNM classifier with them. We also presented their PSI (the Percent Spliced In) values in the 319 LNM negative and 176 LNM positive patients (Fig. 5C). Among the 13 AS events, we discovered two pairs of them (UNG-24277-AP and UNG-24278-AP, CHID1-13807-AT and CHID1-13806-AT) come from the same parent genes, with the intrapair correlation coefficient by Spearman correlation analysis as − 0.9999881 and − 1, respectively. Such AS-based LNM classifier behaved well in both sensitivity and specificity, as its AUC values were in the range of 0.758 to 0.855 in fivefold cross-validation (Fig. 5D). Hence, we verified a reliable AS classifier for the identification of LNM status.

### AS-based prognostic model

**Fig. 6:**
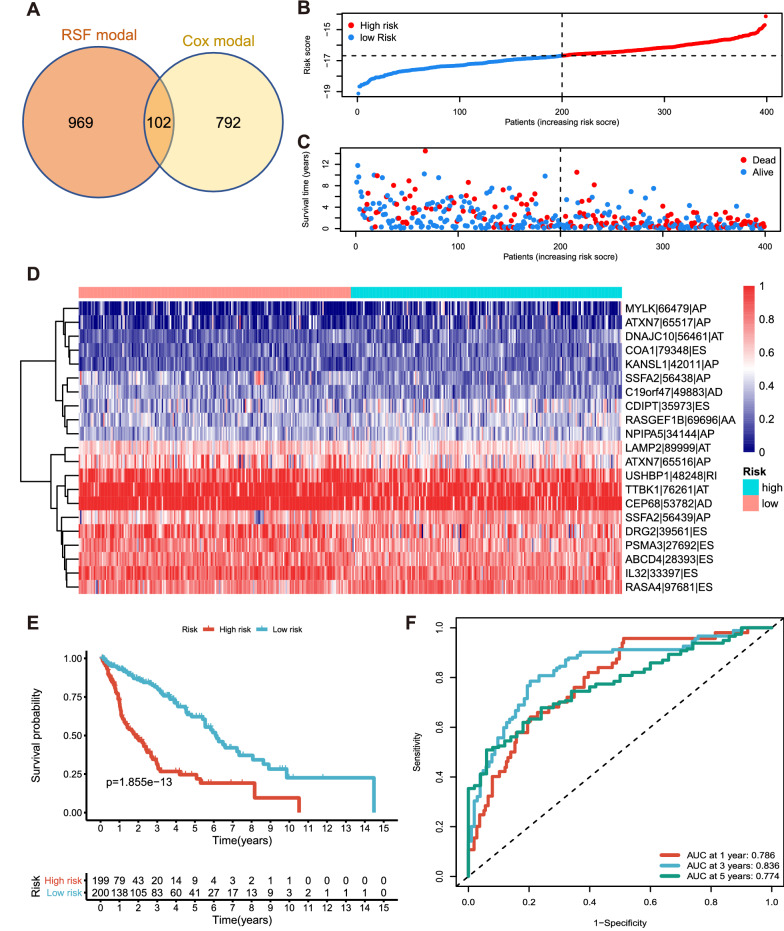
Prognostic model construction and efficiency assessment. **A** Identification of survival-related AS events simultaneously identified by Cox regression and random survival forests. **B**, **C** Visualization of the risk score and survival for each patient. **D** The PSI levels of the 21-ASE signature in the high-risk and the low-risk group. **E** The Kaplan–Meier survival curve for patients in the high-risk and the low-risk group. **F** Time-dependent ROC curves for LUAD patients at 1, 3, and 5 years

To reflect the prognostic prediction ability, we recognized 1071 survival-related AS events by RSF model (Additional file 8: Table S8) and 894 through the Cox regression method (Additional file 9: Table S9), where 102 AS events were presented simultaneously (Fig. 6A). Afterward, the 102 prognosis-associated AS events were included in the forward selection model, and 21 of them were left for the final prognostic risk score model for LUSC via multivariate Cox regression (Additional file 10: Table S10). By the way, two pairs of them (ATXN7-65516-AP and ATXN7-65517-AP, SSFA2-56438-AP and SSFA2-56439-AP) come from the same parent genes, with the intrapair correlation coefficient by Spearman correlation analysis as − 0.6334709 and − 0.9985654, respectively. The patient cohorts were partitioned into low- and high-risk groups according to the median risk score of the prognostic model (Fig. 6B), and an obvious difference in mortality showed between these two groups (Fig. 6C). The PSI levels of related 21 AS events were presented in the heat map (Fig. 6D), and the survival probability of two risk groups was illustrated in the Kaplan–Meier curve (p = 1.855e−013) (Fig. 6E). We plotted ROC curves for the model at 1, 3, and 5 years, and the calculated AUC is 0.786, 0.836, and 0.774, respectively (Fig. 6F).

**Fig. 7:**
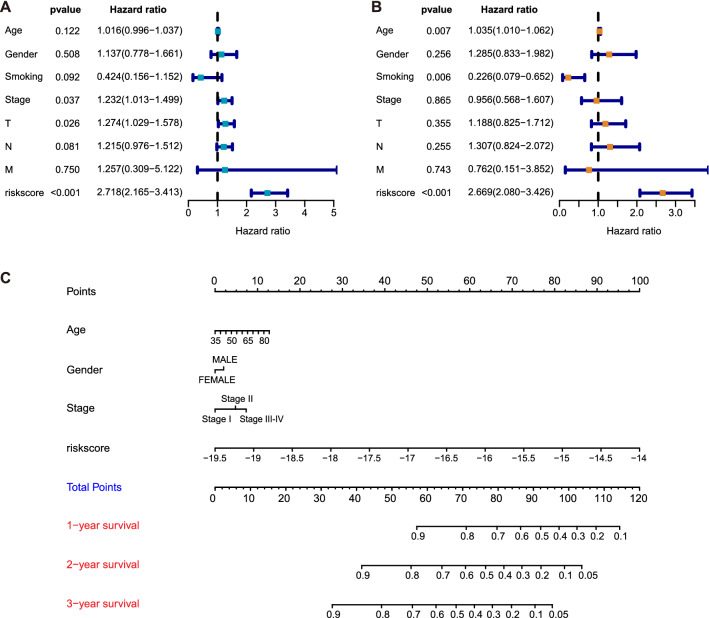
Forest plots and the nomogram for the prognosis of LUSC patients. The evaluation effects of several clinical features and the risk model for the prognostic of LUSC patients assessed by univariate Cox regression analysis (**A**) and multivariate Cox regression analysis (**B)**. **C** The nomogram predicts the overall survival probability of LUSC patients

We analyzed the relationships between the risk score model and clinicopathological factors, then we found there is no significant correlation in American Joint Committee on Cancer (AJCC) stage (P = 0.74), T stage (P = 0.59), N stage (P = 0.16), M stage (P = 0.73), smoking history (P = 0.68), gender (P = 0.28), and age (P = 0.53) except for vital status (P < 0.05) (Additional file 13: Figure S2). Then, these variables introduced above were introduced into univariate and multivariate Cox regression analyses. The risk score showed a significant relationship with overall survival (P < 0.001, both) in two analyses. Besides, in the univariate analysis, the Hazard ratio (HR) of T stage and N stage is 1.232 (95% CI: 1.013–1.499) and 1.274 (95% CI: 1.029–1.578), respectively (Fig. 7A). In the multivariate analysis, the age of patients is presented to be a risk-associated factor (HR = 1.035, 95% CI: 1.010–1.062) while smoking history is a protective factor (HR = 0.226, 95% CI: 0.079–0.652) (Fig. 7B), which could be caused by Simpson’s paradox (as we only compared the smokers with nonsmokers, rather than classifying them based on smoking amount) [27]. A nomogram for prognostic prediction was then developed for clinical application (Fig. 7C). Collectively, we constructed a convenient model for the prognosis of LUSC patients.

### Functional annotation

**Fig. 8:**
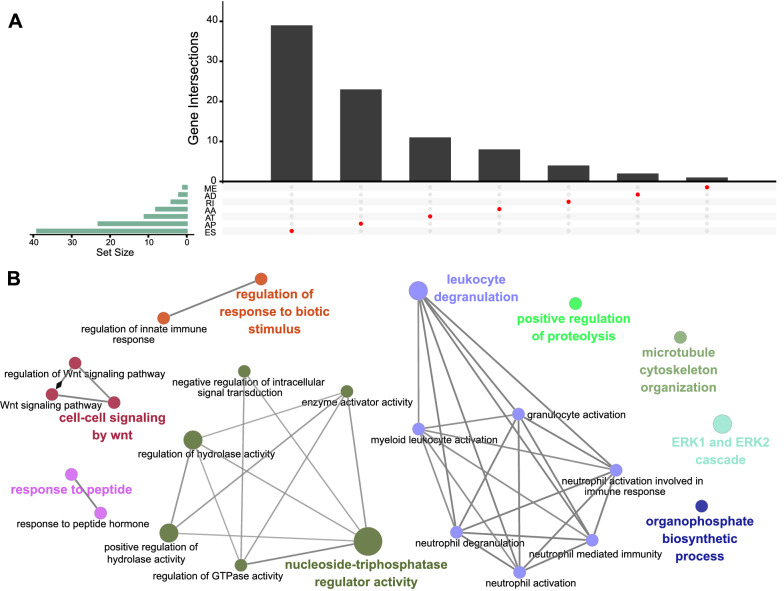
Functional enrichment analyses. **A** The upset plot shows the 102 overlapping AS events selected by Cox regression and random survival forests. **B** Pathway analyses of these genes associated with OS-related splicing events

The 102 identified prognosis-associated AS events were derived from 88 parent genes (Fig. 8A). By utilizing KEGG and Reactome pathway analyses for these parent genes, we enriched pathways associated with prognosis-related splicing events including “nucleoside-triphosphatase regulator activity”, “leukocyte degranulation”, “ERK1 and ERK2 cascade”, “cell–cell signaling by wnt”, “positive regulation of proteolysis”, “regulation of response to biotic stimulus”, and so on (Fig. 8B). Some of these biological pathways have been well-illustrated in human cancers [28–30], whereas their enrichment in splicing processes provides a new perspective for us.

### The splicing-regulatory network

**Fig. 9:**
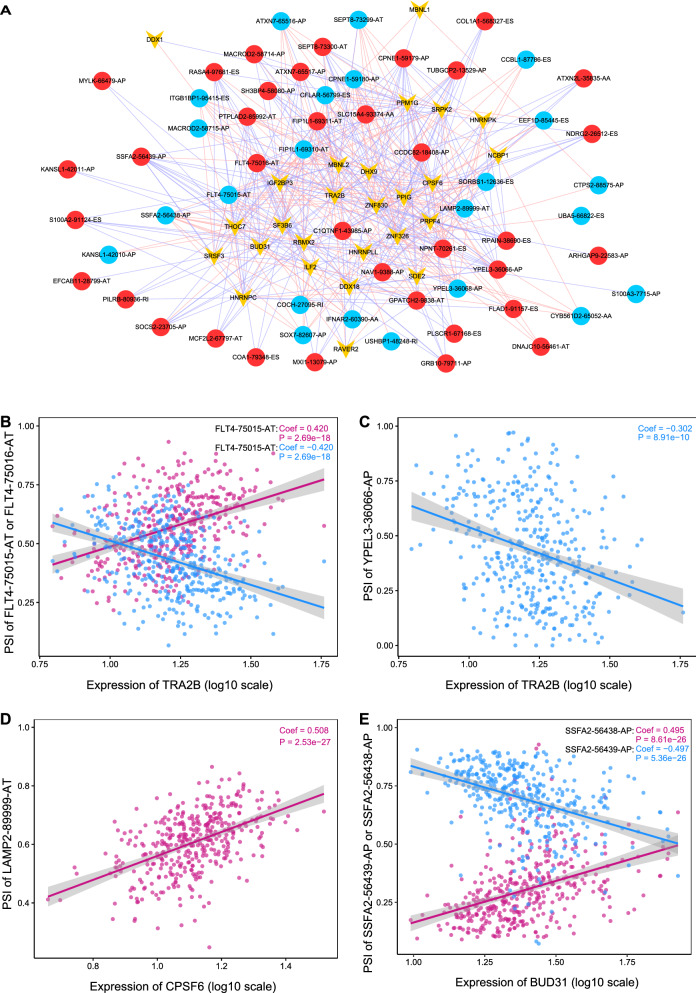
Correlation analysis between splicing factors and AS events in LUSC cohort. **A**The splicing network for splicing factors and AS events. Yellow nodes indicate splicing factors, red nodes indicate poor survival associated with AS events, and blue nodes represent good survival associated with AS events; Red lines represent positive correlation, and blue lines represent the negative correlation. **B** The correlation between PSI values of FLT4-75015-AT or FLT4-75016-AT and the expression of TRA2B. **C** The correlation between PSI values of YPEL3-36066-AP and the expression of TRA2B. **D** The correlation between PSI values of LAMP2-89999-AT and the expression of CPSF6. **E** The correlation between PSI values of SSFA2-56439-AP or SSFA2-56438-AP and the expression of BUD31. The negative correlation is presented in dodger blue, while the positive correlation in medium violate color

After univariate Cox analysis, 26 SFs were selected as survival-associated. The relationships of these SFs and survival-related AS events were calculated and illustrated in a correlation network (Additional file 11: Table S11, Fig. 9A), which contains 36 risk AS events and 23 protective AS events. In these SFs, TRA2B had correlations with the most number of AS events (n = 35), second by CPSF6 (n = 30). In the 59 AS events, 18 of them were paired and spliced from 9 parent genes with different AP or AT patterns, showing opposite correlations when appeared with a same SF. FLT4-75015-AT, FLT4-75016-AT, and YPEL3-36066-AP were under the regulation of the most SFs (n = 15, each). The correlation between the 3 AS events and TRA2B was 0.420, − 0.420, and − 0.302, respectively (Fig. 9B, C). CPSF6 and LAMP2-89999-AT had the strongest correlation as 0.508, followed by correlations between BUD31 and SSFA2-56439-AP (− 0.497) or SSFA2-56438-AP (0.495) (Fig. 9D, E). By introducing upstream SFs for LUSC, we can have a better panoramic view of the AS universe.

## Discussion

Lung squamous cell carcinoma (LUSC) is a common aggressive malignancy arising from lung squamous epithelium and accounts for about one-third of all lung cancer cases. Nowadays, we still have no approved targeted therapies and only depend on surgery, chemotherapy, or radiotherapy for the treatment of LUSC patients [31]. Surgical intervention for patients in the early stage and adjuvant postoperative treatment for those high-risk LUSC patients would truly improve their outcome [32]. Therefore, searching for prognostic and predictive biomarkers is of vital significance and a lot of studies have been performed. For example, by investigating pathological findings, researchers found that tumor budding, single cell invasion, and nuclear diameter have been illustrated as independent prognostic factors for LUSC [33]. A signature based on 5 proteins and a classifier with 2 genes were associated with prognostic outcomes for early-stage LUSC and got verified by a multicenter study, respectively [34, 35]. Some non-coding RNAs, such as hsa_circ_0079530, lnc-IGFBP4-1, and miR-193a-3p, also showed remarkable diagnostic or prognostic potential [36–38].

Over the past decades, along with the rapid development of high-throughput sequencing techniques, the roles of alterations in the splicing process gradually appear [39]. It is well believed that subtype-specific and progression-specific splicing aberrations would usher us into an unprecedented era of cancer studies [16]. Recently, a few studies in LUSC have done some integrative analysis about the prognostic value of AS [40, 41]. However, the comprehensive evaluation of those AS events involved in splicing pattern shifts or the LNM status of LUSC is still lacking. Furthermore, the benefits of random forest-based approaches especially in removing outliers and noise arose our great interest [42, 43]. Here, we took Boruta algorithms and random survival forest into our analysis process, as well as classical Cox regression model and so on. To the best of our knowledge, this study is the first one to systematically analyze AS situations in LUSC patients by utilizing several machine learning methods.

In this study, we determined 16 pairs of completely opposite AS events expressed in LUSC tissues and normal lung tissues. More interestingly, after comparing to those contrarily expressed AS events which we analyzed for LUAD [22], we noticed 5-paired alterations (AP1S2-88569-AT and AP1S2-88571-AT, ITIH5-10716-AT and ITIH5-10715-AT, LDB1-12935-AP and LDB1-12934-AP, PPP3CB-12153-AT and PPP3CB-12154-AT, QKI-78404-AT and QKI-78405-AT) happened in LUSC and LUAD both. One of the shiny parent genes—QKI—was illustrated to be a strong AS regulator in NSCLC [44, 45]. Besides, the shifted splicing pattern of ITIH5 has been introduced to prognosticate the occurrence of colorectal cancers [46], while the altered spicing of PTPN6 was involved in leukemogenesis [47]. These splicing alterations reveal the transitions of protective factors and risk factors, which are potential diagnostic biomarkers and could even be used for therapeutic targets.

We then verified the expression of some AS events and their parent genes by qRT-PCR. Actually, we aimed to calculate the PSI of these AS events by the cycle thresholds (Ct) of our clinical samples as other literature introduced at first [48]. However, then we noticed that the comparison of two different PCR reaction systems with different primers would increase the experimental error, meanwhile, the complexity and heterogeneity of clinical specimens should not be ignored. The classical PSI calculation method is according to the strip gray values of RT-PCR products. But we found what they studied mostly is the ES pattern, so they can design primers by the conservative exons on both sides of the skipped exon and then obtain different PCR products by a single reaction system [49–51]. Absolutely, RT-PCR of exon skipping was demonstrated to have a high accuracy of microarray and high-throughput sequencing results [52, 53]. Whereas, the splicing pattern of switch-associated AS events was all AP or AT in this study. Besides, even though the full sequences of spliced exons and genes were available, it was still difficult to obtain reliable primers for all AS events after several attempts. Therefore, here we verified the relative expression level of parent genes and some of their AS events by qRT-PCR. All the primers were strictly matched with targeted sequences and results were analyzed via the basic local alignment search tool (BLAST) of NCBI, pre-experiments were carried out one by one to obtain optimal qRT-PCR reaction conditions, and the DNA gel electrophoresis and Sanger sequencing were used to check their products. Some of the primers have also been demonstrated in the previous literature such as the primers of QKI [45], ITIH5 [54], and ITIH5-10715-AT [55–58]. What to be mentioned is, the primer of ITIH5-10715-AT in former articles (Forward: 5′-TCACCGTGTGCTTCAACATT-3′; Reverse: 5′-GGGTGCCCCAATTAACTCTC-3′), which can only amplify two out of three INSDC mRNA submissions (with NM_ accession prefixes) and one out of two model RefSeqs (with XM_ accession prefixes) of ITIH5 analyzed by the BLAST of NCBI, was used to test the expression level of ITIH5 genes [55–58], similar with the primer in another research (Forward: 5′-TTCCCGTTATGCCTTCACTAC-3′; Reverse: 5′-TTTCGCCCTGATACACCTTG-3′) [59]. Incomplete amplification products may cause deviations in experimental results, which is what we need to pay enough attention to. Anyway, we illustrated the diagnostic value of ITIH5-10715-AT and QKI-78404-AT in LUSC patients and reflected the existence of splicing switch-associated AS events indirectly. Further research is urged to uncover the underlying machines of such kinds of splicing shifts in cancers.

We also constructed a classifier for the LNM distinction in LUSC. The AUC values in all folds were over 0.75 in fivefold cross-validation, suggesting its high sensitivity and specificity. Among the 13 AS events of our LNM classifier, two pairs coming from the same parent genes both have a strong negative correlation, which reflected the activity of splicing switch in the process of LNM. Considering the lymphatic system is a common invasion target of LUSC and patients with LNM showed a poor outcome [60], we believe it is worthwhile to expend more effort in this area.

For prognostic purposes, firstly, we built a prognostic model by applying the forward selection model into the 102 survival-associated AS events, which were grasped from Cox regression and RSF simultaneously. This scheme was proved to be reliable and simple to implement [22]. Our prognostic model, with 21 AS events, pictured an excellent distinguishing capacity for high-risk and low-risk LUSC patients (P = 1.855e−013), along with a strong predictive ability for survival in 1, 3, and 5 years (AUC > 0.77, each). Next, we analyzed the prognostic potential of clinicopathological factors such as age, gender, and smoking history. Ultimately, we developed a comprehensive predictive model (nomogram) with some OS-related risk factors to improve the sensitivity.

The splicing network presented the importance of some AS regulators. The RNA binding protein TRA2B (SFRS10) is well-established in the AS regulation during biological processes such as somitogenesis and tumorigenesis [61, 62], and its recruitment could influence specific AS patterns like intron retention (IR) [63]. A recent study illustrated the upregulation of TRA2B targeted by miR-335 could promote the cell proliferation of lung cancer [64], another study even verified the TRA2B-DNAH5 fusion as a novel oncogenic driver in LUSC independent to LUAD [65]. We believe that the correlation between TRA2B and survival-related AS events could further explain its function mechanism. CPSF6 could regulate viral alternative RNA processing [66] and the splicing during mouse fetal development [67]. The relationship between CPSF6 and lung cancers is still needed to be revealed. BUD31 is an MYC-synthetic lethal gene and is a potential therapeutic entry point for human breast cancers [68, 69]. The obvious correlation between BUD31 and SSFA2-56439-AP or SSFA2-56438-AP also reflected the importance of paired AS events from the same parent gene in the splicing network.

Actually, there have been some finds noticing the role of AS in LUSC needed to be mentioned specially. Zhao et al. and Li et al. systematically identified the prognostic AS signature in NSCLC, the former group conducted prognostic AS models from the perspective of different sexes [48], and the latter group firstly created prognostic predictors with one type or all types of AS events in LUSC [70]. Yan et al. and Liu et al. also built prognostic models for seven types of AS events one by one recently [40, 41]. In this aspect, our study used combined algorithms and constructed a final prognostic risk score model with a few AS events, which focused on detailed indicators rather than an overview of AS-prognosis significance. What’s more important, we firstly analyzed LUSC-related AS events which implicate splicing switches and identified an AS-based LNM classifier for the first time. Interestingly, paired AS events from the same genes were shown in the selected group of LNM and prognosis biomarkers, revealing the ubiquity and importance of switched AS events in the process of cancer development. Considering different splicing patterns from one parent gene may lead to totally opposite influences on cancer development, researches merely focusing on a single alternative event seems to be unthoughtful. Thus, we comprehensively analyzed the expression of the parent genes and the two splicing isoforms.

Admittedly, there are still some important points in the study that need to be further explored. First, there is only one AS dataset for data analysis and model building, it would be better if another external validation dataset works. Second, it was our regret to not be able to verify the splicing switch-associated AS events directly and integrally due to the limitations of experimental conditions, and the qRT-PCR results may be much more reliable if we could include more research subjects. Third, we have not done interventional experiments for these AS biomarkers yet, further studies are urged to clarify underlying mechanisms.

## Conclusion

In summary, our study found 16 pairs of splicing events strongly related to the splicing switch, built a dependable LNM classifier based on 13 AS events, and developed a prognostic model for LUSC patients according to 21 AS events. We also did functional enrichment analysis and constructed a splicing network to explore AS-associated mechanisms in LUSC. Our findings highlighted the role of paired and switched AS events from a single parent gene. AS events obtained in this study provide new insights into the molecular diagnosis and therapeutic drug design of LUSC.

## Supplementary Information

**Additional file 1: Table S1.** qRT-PCR primers.

**Additional file 2: Table S2.** All samples included in this study.

**Additional file 3: Table S3.** Feature importances and selection results presented by the Boruta algorithm for the splicing events differentiating normal and LUSC tissues.

**Additional file 4: Table S4.** Information of patients involved in the qRT-PCR.

**Additional file 5: Table S5.** Information of splicing switch-associated AS events in LUSC and normal tissues in TCGA SpliceSeq.

**Additional file 6: Table S6.** Raw data of the qRT-PCR.

**Additional file 7: Table S7.** 19 AS events presented by Boruta algorithm for the classifier recognizing lymph node metastasis (LNM).

**Additional file 8: Table S8.** Survival-related alternative splicing events selected by random survival forest model.

**Additional file 9: Table S9.** Unvariate Cox regression results.

**Additional file 10: Table S10.** Alternative splicing events and their coefficient in the prognostic model.

**Additional file 11: Table S11. **The relationships between 26 SFs and selected survival-related AS events.

**Additional file 12: Figure S1 **Supplementary verification of qRT-PCR results. **A** Product sizes of selected primers. **B** Online database verification of qRT-PCR results. For each column (from top to bottom), we presented the expression of parent gene, the PSI values of selected AS event, and the PSI values of another AS event (to be paired with the former one) in normal lung samples and LUSC samples in order. RNA expression data in 49 normal lung tissues and 501 LUSC tissues from the TCGA-LUAD database were replenished for the analysis of LDB1, PTPN6, QKI, and ITIH5. N, normal; C, cancer.

**Additional file 13: Figure S2.** Relationships between clinical features and the risk model. The distribution of risk scores of LUSC patients in different clinical groups. LUSC patients were assigned to different groups according to clinical risk factors. (A) AJCC stage. (B) T stage. (C) N stage. (D) M stage. (E) Vital status. (F) Smoking history. (G) Gender. (H) Age.

## Data Availability

The datasets used and analyzed during the current study are available from the corresponding author on reasonable request.

## Abbreviations

mRNA: Messenger RNA
TCGA: The Cancer Genome Atlas
SCLC: Small cell lung carcinoma
NSCLC: Non-small cell lung carcinoma
LUAD: Lung adenocarcinoma
LUSC: Lung squamous cell carcinoma
OS: Overall survival
ES: Exon skip
AD: Alternate donor site
AA: Alternate acceptor site
RI: Retained intron
ME: Mutually exclusive exons
AT: Alternate terminator
AP: Alternate promoter
PSI: The Percent Spliced In
LNM: Lymph node metastasis
RF: Random forests
AUC: The area under the ROC curve
RSF: Random survival forest
SFs: Splicing factors
qRT-PCR: Quantitative real‐time PCR
AJCC: American Joint Committee on Cancer
HR: Hazard ratio
BLAST: Basic local alignment search tool
