# Supplementary figures and images for: Switched alternative splicing events as attractive features in lung squamous cell carcinoma
Source: Cancer Cell Int. 2022 Jan 5;22:5. doi: 10.1186/s12935-021-02429-2 (PMC8734344; doi:10.1186/s12935-021-02429-2)

**A**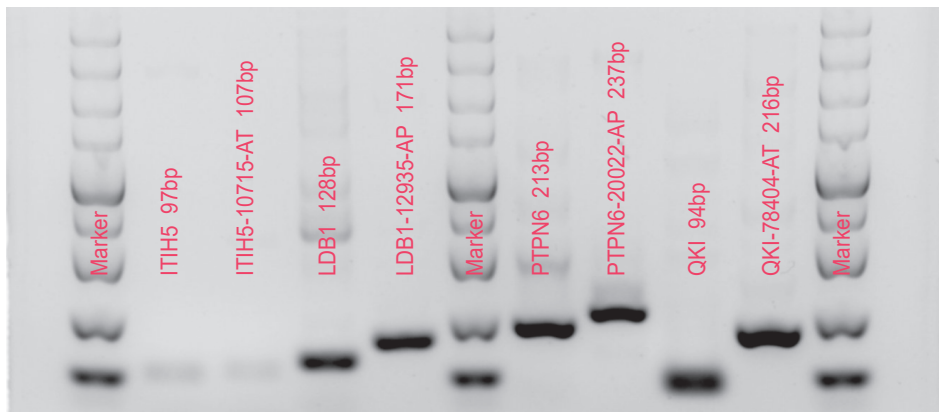**B**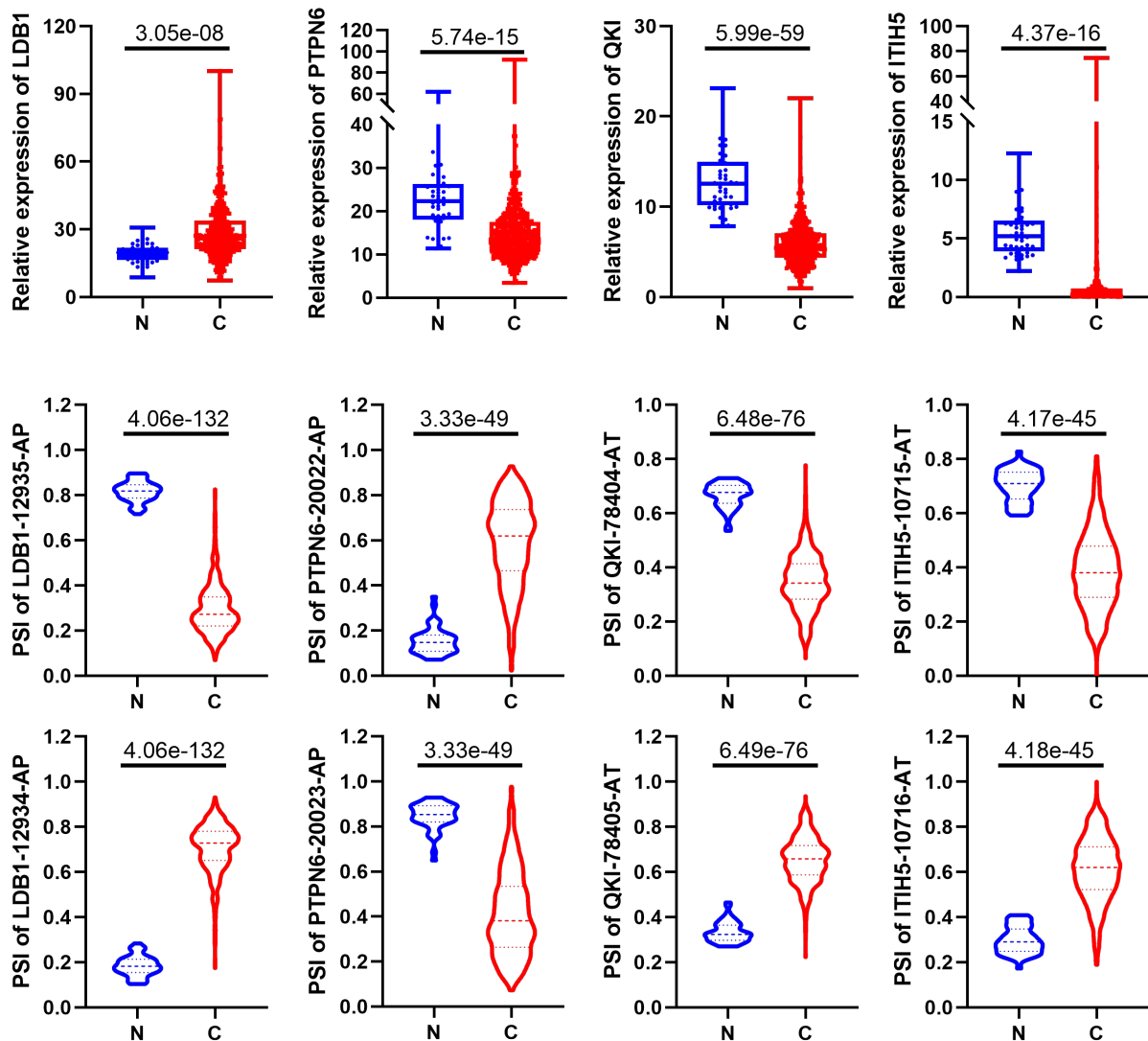

Supplement: Supplementary file 11 — Additional file 11: Table S11. The relationships between 26 SFs and selected survival-related AS events. [file 12935_2021_2429_MOESM11_ESM.pdf]

A

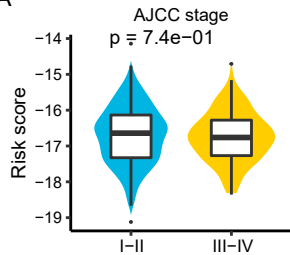

B

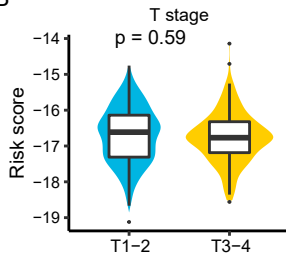

C

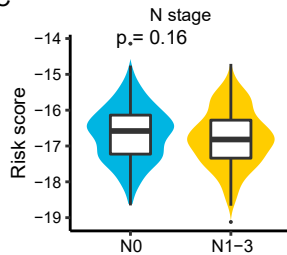

D

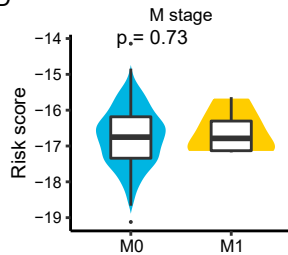

E

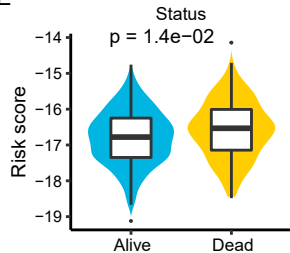

F

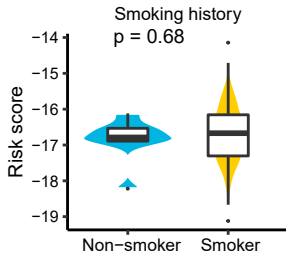

G

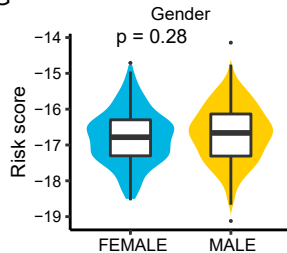

H

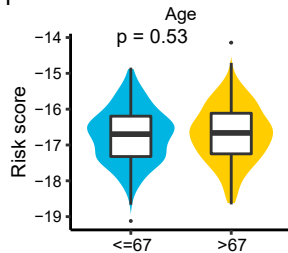

Supplement: Supplementary file 13 — Additional file 13: Figure S2. Relationships between clinical features and the risk model. The distribution of risk scores of LUSC patients in different clinical groups. LUSC patients were assigned to different groups according to clinical risk factors. (A) AJCC stage. (B) T stage. (C) N stage. (D) M stage. (E) Vital status. (F) Smoking history. (G) Gender. (H) Age. [file 12935_2021_2429_MOESM13_ESM.pdf]
